# Replacement of acetate with citrate in dialysis fluid: a randomized clinical trial of short term safety and fluid biocompatibility

**DOI:** 10.1186/1471-2369-14-216

**Published:** 2013-10-09

**Authors:** Gunilla Grundström, Anders Christensson, Maria Alquist, Lars-Göran Nilsson, Mårten Segelmark

**Affiliations:** 1 Gambro Research, Gambro Lundia AB, Lund, Sweden; 2 Department of Nephrology and Transplantation, Skåne University Hospital, Lund, Sweden; 3 Gambro Medical Safety Office, Gambro Lundia AB, Lund, Sweden; 4 Department of Medicine and Health, Linköping University, Linköping, Sweden

## Abstract

**Background:**

The majority of bicarbonate based dialysis fluids are acidified with acetate. Citrate, a well known anticoagulant and antioxidant, has been suggested as a biocompatible alternative. The objective of this study was to evaluate short term safety and biocompatibility of a citrate containing acetate-free dialysis fluid.

**Methods:**

Twenty four (24) patients on maintenance dialysis three times per week, 13 on on-line hemodiafiltration (HDF) and 11 on hemodialysis (HD), were randomly assigned to start with either citrate dialysis fluid (1 mM citrate, 1.5 mM calcium) or control fluid (3 mM acetate, 1.5 mM calcium) in an open-labeled cross-over trial (6 + 6 weeks with 8 treatments wash-out in between). Twenty (20) patients, 11 on HDF and 9 on HD were included in the analyses. Main objective was short term safety assessed by acid–base status, plasma ionized calcium and parathyroid hormone (PTH). In addition, biocompatibility was assessed by markers of inflammation (pentraxin 3 (PTX-3), CRP, IL-6, TNF-α and IL-1β) and thrombogenicity (activated partial thromboplastin time (APTT) and visual clotting scores).

**Results:**

No differences dependent on randomization order or treatment mode (HD vs. HDF) were detected. Citrate in the dialysis fluid reduced the intra-dialytic shift in pH (+0.04 week 6 vs. +0.06 week 0, p = 0.046) and base excess (+3.9 mM week 6 vs. +5.6 mM week 0, p = 0.006) over the study period. Using the same calcium concentration (1.5 mM), citrate dialysis fluid resulted in lower post-dialysis plasma ionized calcium level (1.10 mM vs. 1.27 mM for control, p < 0.0001) and higher post-dialysis PTH level (28.8 pM vs. 14.7 pM for control, p < 0.0001) while pre-dialysis levels were unaffected. Citrate reduced intra-dialytic induction of PTX-3 (+1.1 ng/ml vs. +1.4 ng/ml for control, p = 0.04) but had no effect on other markers of inflammation or oxidative stress. Citrate reduced visual clotting in the arterial air chamber during HDF (1.0 vs. 1.8 for control, p = 0.03) and caused an intra-dialytic increase in APTT (+6.8 s, p = 0.003) without affecting post-dialysis values compared to control.

**Conclusions:**

During this small short term study citrate dialysis fluid was apparently safe to use in HD and on-line HDF treatments. Indications of reduced treatment-induced inflammation and thrombogenicity suggest citrate as a biocompatible alternative to acetate in dialysis fluid. However, the results need to be confirmed in long term studies.

**Trial registration:**

ISRCTN: ISRCTN28536511

## Background

Reintroduction of bicarbonate as the main buffer in dialysis fluids in the late 1980’s revolutionized hemodialysis, but since then no major improvements in dialysis fluid composition have been made. Preparation of bicarbonate based dialysis fluids requires an acidic component in order to balance pH and most commonly acetic acid is used in a concentration of 3–10 mM. This is up to a hundred times higher than normal plasma acetate levels and typically results in a substantial increase in plasma acetate which may promote hemodynamic instability, inflammation, and acidosis [1-3]. Citric acid has been proposed as a more biocompatible acidifier being a natural metabolite and antioxidant with anti-inflammatory properties.

Metabolic acidosis of chronic kidney disease is associated with protein wasting and malnutrition and can cause cardiovascular instability due to impaired myocardial contractility [4,5]. Unlike acetate, citrate is rapidly metabolized into carbon dioxide and energy. Clinical studies have shown that citrate containing dialysis fluids can improve both the acidotic and nutritional state in patients compared to acetate containing fluids [6-8].

Cardiovascular disease (CVD) is the most common cause of death among dialysis patients with a 10–20-fold higher mortality rate than in the general population [9-11]. Inflammation and oxidative stress have been identified as specific risk factors for developing CVD in dialysis patients and are proposed as potential therapy targets [10,12-14]. By complex binding calcium citrate inhibits both coagulation and complement activation and may reduce the treatment-induced inflammatory response [15,16]. Citrate also reduces oxidative stress by chelating multivalent cations like iron and copper and has been shown to restore mitochondrial glutathione levels and decrease markers of cellular injury and free radical formation [17-19]. Apart from causing inflammation blood activation may also reduce dialysis efficiency by inducing clogging of the dialyzer. Clinical studies have shown improved clearance when using a citrate containing dialysis fluid compared to a regular dialysis fluid [20,21].

The aim of this randomized controlled cross-over pilot study was to investigate short term safety and biocompatibility of a citrate containing acetate free dialysis fluid during hemodialysis (HD) and post-dilution hemodiafiltration (HDF). Safety was assessed by patient acid base status and calcium balance (plasma ionized calcium and PTH levels). Fluid biocompatibility was assessed by various markers of inflammation, oxidative stress and coagulation activation. In addition, potential citrate accumulation, hemodynamics and treatment efficiency were investigated.

## Methods

### Patients and study design

**Figure 1:**
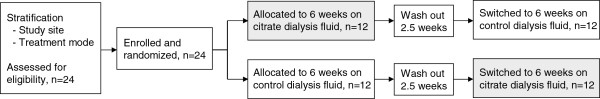
Study design flow diagram.

The study was conducted in accordance with the International Standard ISO 14155, and ethical principles, derived from the Declaration of Helsinki and approved by the regional ethical review board of Lund University. All patients were legally adults and signed an informed consent to participate in the study prior to any study related procedures. The study was a randomized controlled cross-over study with a wash-out period, Figure 1. Twenty-four stable patients (16 male and 8 female) undergoing maintenance hemodialysis three times per week at the Skåne University Hospital in Lund and Malmö, Sweden, were recruited and enrolled. Patients were stratified according to dialysis centre (16 in Lund and 8 in Malmö) and treatment mode (13 on HDF and 11 on HD). Patients were randomized to treatment thrice weekly for six weeks with either the citrate containing dialysis fluid or the control acetate containing dialysis fluid. After a wash-out period (eight consecutive treatments with control fluid) patients switched fluid for another six weeks. The start and end of each study period and all data collection and blood sampling occurred on mid week treatments. Randomization was performed using block randomization with site (Lund or Malmö) and treatment mode (HD or HDF) as stratification variables. Randomized allocation occurred after enrollment and was conducted by an independent CRO. First patient in occurred 13^th^ of January 2011 and last patient out 19^th^ of May 2011.

The study was performed using the Gambro dialysis monitor (AK 200™ ULTRA S) and Gambro single-use high flux dialyzers (Polyflux™ 170H or Polyflux™ 210H). Both the citrate containing A-concentrate (SelectBag® Citrate) and the control (SelectBag® One) were used together with sodium bicarbonate (BiCart®) and sodium chloride (SelectCart®) containing cartridges. For final dialysis compositions see Table 1. Dialysis fluid flow rate was kept constant at 500 ml/min.

**Table 1:** Dialysis fluid compositions

**Compound**	**Citrate fluid**	**Control fluid**
Potassium (mM)	2 or 3	2 or 3
Magnesium (mM)	0.5	0.5
Calcium (mM)	1.5	1.5
Sodium (mM)	Variable	Variable
Bicarbonate (mM)	34-36	34-36
Glucose (mM)	5.5	5.5
Citrate (mM)	1	0
Acetate (mM)	0	3

Anticoagulation was performed using low molecular weight heparin (N = 23; single bolus dose) or unfractionated heparin (N = 1; initial bolus + second bolus). Anticoagulation prescriptions and concomitant medications were unchanged during the study period. Blood pressure, heart rate, blood flow rate, ultrafiltration rate and extracorporeal arterial and venous pressures were recorded. Treatment efficiency was measured as ionic Kt/V (Diascan® monitoring tool). Clotting degree in dialyzers, venous drip chambers and arterial expansion chambers were rated by qualified nurses according to validated scales. For venous and arterial chambers no clotting was rated as 0 and complete clotting as 4. For dialyzers no clotting was scored as 0 and complete clotting as 3.

### Laboratory analyses

Arterial blood samples were taken pre- and post-dialysis at the mid-week dialysis sessions. Safety parameters (ionized calcium, PTH, citrate, hemodynamics and acid base parameters) were measured every second week and markers of coagulation, inflammation and oxidative stress were measured at the beginning and end of each study period. Plasma was separated and kept frozen at −80°C if not analyzed immediately.

The following routine samples were analyzed at the Department of Clinical Chemistry Skåne University Hospital: blood erythrocyte, leukocyte and thrombocyte counts, hemoglobin, hematocrit, sodium, potassium, chloride, total and ionized calcium, parathyroid hormone (PTH), urea, albumin, pH, carbon dioxide pressure (pCO_2_) and base excess. Inflammatory markers C-reactive protein (CRP), interleukin 6 (IL-6), interleukin 1β and tumor necrosis factor α were analyzed on site by standard methods. Activated partial thromboplastin time (APTT) was assayed optically with equipment routinely applied at the study sites.

Citrate plasma levels were analyzed in heparinized plasma with a validated chromatographic (HPLC) method developed by Gambro Research, Lund. Total advanced glycation end-products were determined spectrophotometrically and carboxymethyl-lysine and pentosidine by commercial ELISA kits (Cell Biolabs, Inc. USA) at Gambro Research, Lund.

Several biocompatibility parameters were analyzed at the Department of Laboratory Medicine at Karolinska University Hospital in EDTA-treated plasma. Pentraxin 3 (PTX-3) was analyzed by a commercially available ELISA kit (Perseus Preteomics Inc., Japan). Total aminothiols were analyzed by a validated HPLC method, modified advanced oxidation protein products by a colometric bioassay and 8-oxo-2′-deoxyguanosine by a competitive ELISA kit (Japan Institute for Control of Aging, Shizuoka, Japan).

### Statistics

Analyses were done using standard analysis of covariance for a two-period cross over study. Within patient difference between the two periods, divided by treatment mode (HD/HDF) and study site, were analyzed using the Wilcoxon Signed Rank Sum test. Period and carry-over effects were non-significant so the within patient difference between the two periods were used for analyses. Data in tables and graphs are presented as means ± standard error of mean (SEM) unless otherwise stated. All statistical tests are interpreted at the 5% significance level (2-tailed). Sub-analyzes power calculations were done using nQuery Advisor® 7.0 based on two group t-test (paired) of equal means.

## Results

### Study population characteristics and statistics

Twenty-four patients on maintenance hemodialysis three times per week were included and entered the study, 13 treated with HDF and 11 with HD. One patient died during a planned angiography and three more were excluded from per protocol analysis due to protocol deviations. Data from a total of 20 patients (11 on HDF and 9 on HD) were included in the per protocol analysis and presented in this paper. No differences between treatment modes (HD/HDF) were detected and all data presented include both treatment modes. No carry over effects were detected and data from both study arms are combined. Patient characteristics are presented in Table 2 and adverse events in Additional file 1: Table S1.

**Table 2:** Study population characteristics at inclusion

	**Lund clinic**		**Malmö clinic**		**Total**
**Parameter, Mean ± StDev**	**HD**	**HDF**	**HD**	**HDF**	**Mean (range)**
Gender (female : male)	2 : 5	3 : 6	2 : 2	1 : 3	8 : 16
Age (years)	74.3 ±11.1	73.9 ±7.6	61.7 ±20.6	59.8 ±15.7	70 (40-92)
Dry weight (kg)	71.9 ±10.6	80.0 ±13.2	77.4 ±25.5	80.4 ±12.2	77 (53-103)
Time on dialysis (years)	2.0 ±2.7	3.2 ±2.1	1.4 ±1.2	8.3 ±11.9	3.4 (0.2-26)

### Acid-base status

**Figure 2:**
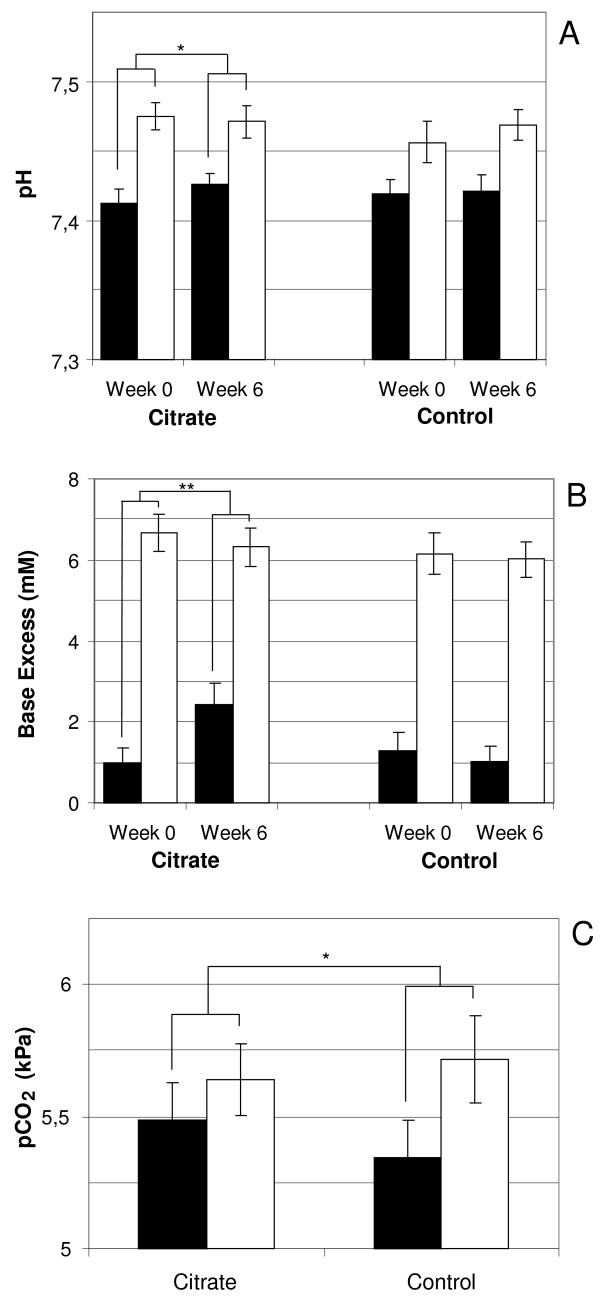
**Impact of citrate dialysis fluid on acid–base status. A)** Intra-dialytic change in pH at study start (Week 0) and study end (Week 6). **B)** Intra-dialytic change in Base Excess at study start (Week 0) and study end (Week 6). **C)** Overall intra-dialytic change in carbon dioxide pressure when using citrate dialysis fluid compared to control. Black bars represent pre-dialysis values and white bars post-dialysis values. Bars show means and error bars represent SEM, n = 20. * = p < 0.05 and ** = p < 0.01.

Citrate dialysis fluid significantly reduced the intra-dialytic pH shift in arterial blood over the six week study period (+0.04 ±0.01 at week 6 vs. +0.06 ±0.01 at week 0, p = 0.046), associated with a trend towards increased pre-dialysis pH, Figure 2A. Citrate dialysis fluid also induced a small but significant increase in post-dialysis pH (7.47 ±0.01 vs. 7.46 ±0.01 for control, p = 0.02) however, no trend towards an increase over time was seen.

During the citrate dialysis fluid period the intra-dialytic base excess shift was significantly reduced (+3.9 ±0.5 mM at week 6 vs. +5.6 ±0.5 mM at week 0, p = 0.006) and after 6 weeks it was significantly lower compared to control (+3.9 ±0.5 vs. +5.0 ±0.5 mM, p = 0.02), Figure 2B. The effect was associated with a significant increase in pre-dialysis base excess during the citrate study period (2.4 ±0.5 mM at week 6 vs. 1.0 ±0.4 mM at week 0, p = 0.03). Citrate dialysis fluid resulted in a small but significant increase in post-dialysis base excess (6.4 ±0.4 vs. 6.0 ±0.5 mM for control, p = 0.04) but no trend towards an increase over time was detected.

Intra-dialytic shift in carbon dioxide pressure (pCO_2_) was significantly lower when using citrate dialysis fluid compared to control (+0.16 ±0.09 vs. +0.39 ±0.14 kPa, p = 0.02), Figure 2C. The effect was associated with a significant increase in pre-dialytic pCO_2_ (5.48 ±0.15 vs. 5.34 ±0.15 kPa for control, p = 0.04) while post-dialysis pCO_2_ were similar between products (5.64 ±0.14 vs. 5.72 ±0.17 kPa for control, ns), Figure 2C. Acid-base parameters were measured pre- and post-dialysis every second week (week 0, 2, 4 and 6) during each study period. Power analyses of reduced intra-dialytic change in pH and base excess at the end of the citrate period gives a power of approximately 30 and 50% respectively.

### Calcium balance and regulation

**Figure 3:**
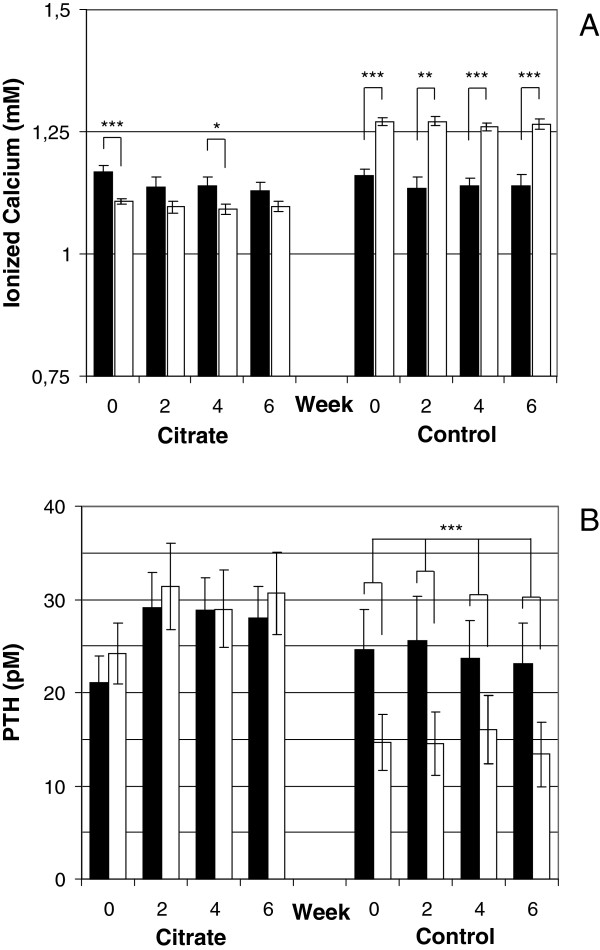
**Analyses of calcium balance over the study period. A)** Intra-dialytic change in plasma ionized calcium levels at each sampling occasion when using citrate dialysis fluid compared to control. **B)** Intra-dialytic change in plasma PTH levels at each sampling occasion when using citrate dialysis fluid compared to control. Black bars represent pre-dialysis values and white bars post-dialysis values. Bars show means and error bars represent SEM, n = 20. * = p < 0.05, ** = p < 0.01 and *** = p < 0.001 and ** = p < 0.01.

With 1.5 mM calcium in the dialysis fluid the presence of 1 mM citrate induced a small but significant intra-dialytic reduction in ionized calcium (1.14 ±0.02 mM pre-dialysis vs. 1.10 ±0.01 mM post-dialysis, p < 0.0001) while there was a significant increase in post-dialysis calcium with the control fluid (1.14 ±0.02 mM pre-dialysis vs. 1.27 ±0.01 mM post-dialysis, p < 0.0001), Figure 3A. Pre-dialysis levels of ionized calcium did not differ between products, or over time, and total calcium levels were constant. Ionized calcium was measured pre- and post-dialysis every second week (week 0, 2, 4 and 6) during each study period while total calcium was analyzed pre-dialysis at the beginning and end (week 0 and week 6) of each period.

With citrate dialysis fluid there was no significant intra-dialytic shift in PTH levels (26.7 ±3.4 pM pre-dialysis vs. 28.8 ±4.1 pM post-dialysis, ns) while the control fluid induced a significant decrease in post-dialysis PTH levels (24.3 ±4.3 pM pre-dialysis vs. 14.7 ±3.3 pM post-dialysis, p < 0.001), Figure 3B. Pre-dialysis levels of PTH did not differ between products. PTH levels were measured pre- and post-dialysis every second week (week 0, 2, 4 and 6) during each study period.

### Citrate tolerance

**Figure 4:**
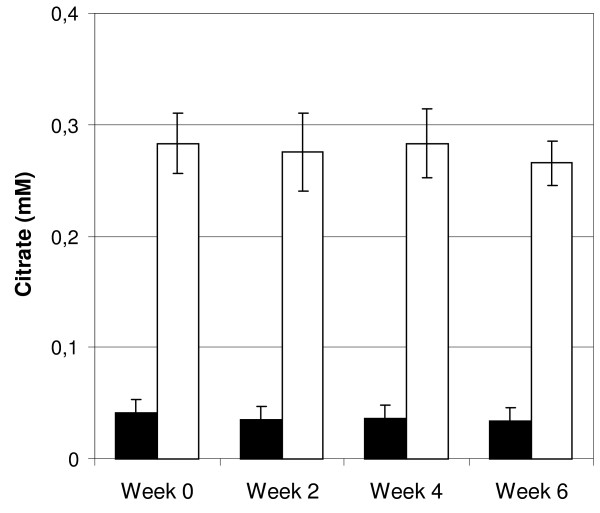
**Effect on plasma citrate levels over the citrate dialysis fluid study period.** Black bars represent pre-dialysis values and white bars post-dialysis values. Bars show means and error bars represent SEM, n = 20.

Citrate plasma levels increased significantly during dialysis with citrate containing dialysis fluid (0.04 ±0.01 pre-dialysis vs. 0.28 ±0.03 mM post-dialysis, p < 0.0001). Citrate levels returned to baseline between treatments and no trend towards accumulation could be detected, Figure 4. Citrate plasma levels were not affected during the control period (data not shown). Citrate levels were measured pre- and post-dialysis every second week (week 0, 2, 4 and 6) during each study period.

The need for medical staff intervention did not differ between periods and no product related adverse event or significant side effects were reported (Additional file 1: Table S1). Blood count parameters; leucocytes, erythrocytes, thrombocytes, hemoglobin and hematocrit, were all within normal variations and did not differ between products.

### Inflammation

**Figure 5:**
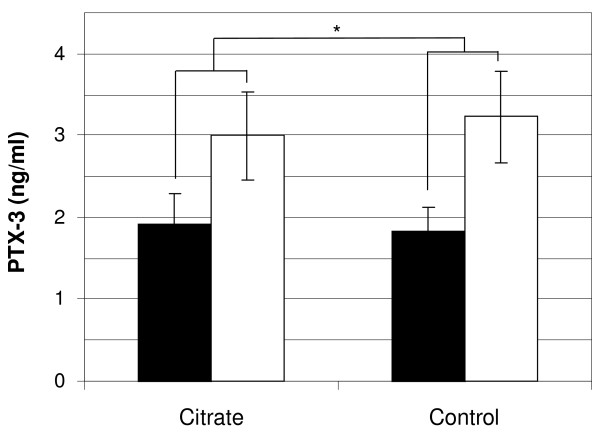
**Effect of citrate dialysis fluid on inflammation presented as overall intra-dialytic change in PTX-3 expression when using citrate dialysis fluid compared to control.** Black bars represent pre-dialysis values and white bars post-dialysis values. Bars show means and error bars represent SEM, n = 20. * = p < 0.05.

Citrate dialysis fluid induced a significantly lower intra-dialytic rise in PTX-3 compared to control (+1.1 ±0.3 vs. +1.4 ±0.3 ng/ml, p = 0.04), suggesting less treatment-induced inflammation, Figure 5. Pre-dialysis values of PTX-3 were not significantly different between products (1.9 ±0.4 vs. +1.8 ±0.3 ng/ml for control, ns). Citrate’s ability to reduce the intra-dialytic increase in PTX-3 compared to control rendered 99% power.

No differences were detected between the two study products regarding plasma levels of other markers of inflammation, including oxidative stress markers and AGEs, Additional file 1: Table S2. All inflammatory markers were measured at beginning and end (week 0 and week 6) of each study period.

### Thrombogenicity

Citrate dialysis fluid induced a significant intra-dialytic increase in APTT (36.6 ±1.9 s pre-dialysis vs. 43.4 ±3.4 s post-dialysis, p = 0.003) not seen with the control fluid (38.1 ±2.4 s pre-dialysis vs. 40.6 ±2.3 s post-dialysis, ns). Apart from the intra-dialytic increase there were no significant differences in APTT between periods. APTT was measured at beginning and end of each study period. Citrate’s small but significant effect on APTT compared to control rendered 99% power.

The arterial expansion chamber during HDF showed significantly lower clotting score after dialysis with citrate dialysis fluid compared to control (0.9 ±0.2 vs. 1.6 ±0.3, p = 0.03). Inspection of the venous drip chamber did not show any significant differences between citrate dialysis fluid and control (1.5 ±0.3 vs. 1.8 ±0.3, ns) and neither did inspection of the dialyzer (0.6 ±0.2 vs. 0.6 ±0.2, ns).

### Hemodynamics and treatment efficiency

**Figure 6:**
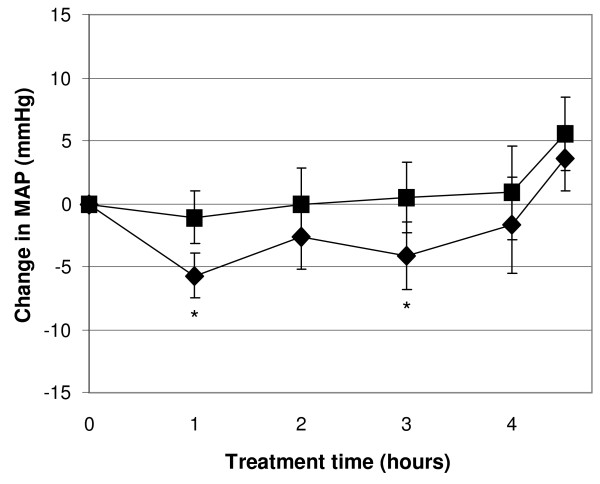
**Changes in mean arterial blood pressure during the dialysis treatment.** Diamonds represent citrate dialysis fluid and squares control dialysis fluid. Symbols show means and error bars represent SEM, n = 20, * = p < 0.05.

Pre-dialysis mean arterial pressure (MAP) was similar between products. Dialysis treatment with citrate dialysis fluid was associated with a lower MAP after 1 h compared to control (90.4 ±2.9 vs. 93.1 ±3.0 mmHg for control, p = 0.03). Relative to pre-dialysis value MAP tended to be lower throughout the treatment when using citrate dialysis fluid compared to control fluid, Figure 6. There were no differences in heart rate between study products and no product related hypotension episodes were reported (data not shown). Hemodynamic parameters were recorded every second week (week 0, 2, 4 and 6) during each study period.

Mid-dialysis ionic Kt/V was significantly higher using citrate dialysis fluid compared to control (0.76 ±0.04 vs. 0.69 ±0.03 for control, p = 0.007) but there was no significant difference in final Kt/V (1.49 ±0.06 vs. 1.47 ±0.07 for control, ns), Table 3. No differences in plasma levels of sodium, potassium, chloride, urea and albumin were detected (data not shown). Dialysis efficiency was analyzed at the beginning and end (week 0 and week 6) of each study period.

**Table 3:** Study treatment characteristics described as mean post-dialysis values, (n = 20)

**Parameter, Mean ± StDev**	**Citrate fluid**	**Control fluid**	**p-value**
Treatment time (h)	4-5	4-5	ns
QB (ml/min)	324 ±35	321 ±35	ns
UF rate (ml/h)	438 ±288	513 ±306	ns
Substitution volume if HDF (l), n = 11	28.2 ±5.1	27.7 ±6.6	ns
Diascan Kt/V	1.49 ±0.26	1.47 ±0.29	ns
Systolic BP (mmHg)	146 ±25	146 ±26	ns
Diastolic BP (mmHg)	76 ±12	74 ±12	ns
Pulse (bpm)	78 ±15	76 ±12	ns

## Discussion

Apart from the introduction of ultrapure fluids no major steps towards more physiological dialysis fluids have been implemented since the introduction of bicarbonate based dialysis in the 1980’s [22,23]. In addition, the possibility to improve tolerance to treatment by adding biologically active substances to the dialysis fluid has only recently been addressed. In this small sized randomized controlled pilot study we addressed potential benefits, as well as short term safety, of an acetate-free dialysis fluid containing 1 mM of citrate.

The most notable finding in this study was the change in patient acid-base status, reflected by both reduced intra-dialytic shifts and increased pre-dialysis steady state levels of pH as well as base excess. Citrate dialysis fluid rendered higher pre-dialysis levels of base excess, carbon dioxide pressure and pH, a combination that indicates reduced metabolic acidosis [24]. Clinical effects of citrate dialysis fluids on acid–base status have been reported before. A short term cross-over study with alternating weeks of citrate (0.8 mM) and regular dialysis fluid showed increased pre-dialysis bicarbonate levels but unexpectedly lower post-dialysis bicarbonate and pH with citrate [25]. However, the study was not designed to detect changes over time. Another study reported improved pre-dialysis steady state bicarbonate and pH after three months with citrate dialysis fluid (0.67 mM) compared to an acetate dialysis fluid [8]. In this study however, the control fluid contained 8 mM acetate and bicarbonate settings differed between citrate and control fluid (35 mM vs. 25 mM) making it hard to evaluate the effect of citrate. A more efficient management of patient acid–base status, where intra- and inter-dialytic fluctuations are avoided, could reduce treatment-induced discomfort and improve patient well-being [4]. Today acidosis is routinely managed by loading the patients with non-physiological levels of bicarbonate during dialysis. Citrate in the dialysis fluid may help reducing acidosis and for patients who do not suffer from acidosis, like our study population, citrate may provide an opportunity to make dialysis more physiological by reducing the bicarbonate load. During our study each patient received the same prescription of bicarbonate throughout the study (34-36 mM).

Our short term study was not optimized for measuring long term effects of citrate dialysis fluid on inflammation but we observed signs of reduced treatment-induced inflammation. The intra-dialytic rise in PTX-3 was significantly lower with citrate compared to control. PTX-3 is associated with cardiovascular morbidity in HD patients and shown to be induced by the dialysis treatment [26,27]. Even though not seen in our short term study citrate dialysis fluid (0.67 mM) used for three months has been shown to reduce pre-dialysis levels of CRP and IL-6 [8]. The antioxidative effect of citrate has also been confirmed *in vitro* as reduced endothelial apoptosis and neutrophil activation under hyperglycemic conditions [17]. Furthermore, the use of citrate as regional anticoagulant during hemodialysis reduced post-dialysis oxidative stress (ox-LDL) and decreased leukocyte degranulation and platelet activation during dialysis [28].

Citrate forms complexes with calcium and a potential concern with citrate dialysis fluids is its effect on systemic ionized calcium. In this study we chose the same calcium concentration (1.5 mM) in both test and control fluid to be able to evaluate the effect of citrate on calcium balance. Under these circumstances citrate caused a small reduction in post-dialysis ionized calcium with a concomitant increase in post-dialysis PTH compared to control. However all changes were within normal ranges and no episodes of hypocalcaemia were reported. In addition, pre-dialysis levels of both PTH and calcium were similar between products. Since part of the citrate-calcium complexes formed in the blood are removed during dialysis citrate dialysis fluids will create a net loss of calcium compared to traditional fluids with the same calcium concentration. However, the actual loss is not as big as indicated by plasma levels of ionized calcium directly after dialysis since calcium will be released from the complexes when citrate is metabolized.

Low ionized calcium concentrations may reduce myocardial contractility and contribute to hypotension episodes during dialysis. We noticed a small reduction in MAP during dialysis but there were no indications of symptomatic hypotension episodes. The reduction in MAP was evident one hour after treatment start which may reflect an initial lowering in plasma ionized calcium. Similar results were seen in a randomized controlled cross-over study where an increase in calcium (1.5 vs. 1.25 mM) in the citrate dialysis fluid (0.8 mM) increased systolic blood pressure and stroke volume while blood peripheral resistance was reduced [29]. However, work by the same group showed that a citrate dialysis fluid (0.8 mM) decreased blood peripheral resistance independent of calcium levels, indicating another mechanism besides calcium signaling [25]. Today the risk of high calcium levels and its role in adynamic bone disease (ADB) and arterial calcification is as debated as the risk of low calcium levels, and a high calcium load in combination with suppressed PTH expression has been associated with arterial stiffness [30,31]. There is also a link between inflammation and oxidative stress and hypertension [32].

We have experienced a general concern about the citrate load during HDF, especially during post-dilution fluid replacement. However, mathematically it has been established that the solute transport across a dialysis membrane is dependent on clearance and not mode of treatment. Even though clearance is generally slightly higher during post-dilution HDF than during HD, the difference is small and can be compensated by an increase in flow rates and/or the membrane permeability during HD [33]. Our study showed in practice that the apparent citrate load and change in ionized calcium was independent of whether the patient was treated with HD or HDF.

In our study we observed signs of reduced treatment-induced thrombogenicity with citrate dialysis fluid. Systemically we noticed an intra-dialytic increase in APTT and in the extracorporeal circuit there was significantly lower clotting score in the arterial air chamber during HDF. Even though not supported by our study, an increase in clearance that may be explained by less clotting in the dialyzer has been reported with citrate dialysis fluid (0.8 mM) [20]. Some publications claim an ability to reduce the heparin dose with citrate dialysis fluid (0.8 mM) but in general these studies are not controlled [21]. Citrate dialysis fluid may be an alternative or complement in certain high risk patients but in order to make a general statement or recommendation about heparin reduction large controlled studies are needed.

## Conclusion

To conclude, citrate dialysis fluid was a safe alternative during this short term study for both HD and on-line HDF treatments. Citrate dialysis fluid reduced intra-dialytic changes in acid–base status by improving pre-dialysis values of both pH and base excess. In addition citrate dialysis fluid was associated with reduced treatment-induced inflammation (PTX-3) and thrombogenicity (APTT). Although our data need to be confirmed in a larger study population over longer time, this randomized controlled study indicates that citrate dialysis fluid may be a more biocompatible alternative to conventional acetate containing fluids.

### CONSORT guidelines

This study is reported following the CONSORT guidelines for randomized controlled trials, Additional file 2: Table S3 [34,35].

## Competing interests

G Grundström, M Alquist and L-G Nilsson are employed by the study sponsor Gambro Lundia AB.

## Authors’ contributions

GG was the main author, involved in the study design and responsible for data interpretation. AC is one of the study investigators and medical experts and revised and approved the final manuscript. MA served as one of the medical experts and revised and approved the final manuscript. L-GN was involved in data interpretation and revised and approved the final manuscript. MS was the principle investigator of the study, medical expert and revised and approved the final manuscript. All authors read and approved the final manuscript.

## Pre-publication history

The pre-publication history for this paper can be accessed here:

http://www.biomedcentral.com/1471-2369/14/216/prepub

## Supplementary Material

Additional file 1: Table S1 Adverse events. **Table S2.** Biocompatibility parameters. Click here for file

Additional file 2: Table S3 CONSORT 2010 checklist of information to include when reporting a randomised trial. Click here for file
