# Supplementary material for: Replacement of acetate with citrate in dialysis fluid: a randomized clinical trial of short term safety and fluid biocompatibility
Source: BMC Nephrol. 2013 Oct 9;14:216. doi: 10.1186/1471-2369-14-216 (PMC4124727; doi:10.1186/1471-2369-14-216)
Supplement: Additional file 1: Table S1 — Adverse events. Table S2. Biocompatibility parameters. [file 1471-2369-14-216-S1.pdf]

TABLE S1: Adverse events

**Summary of adverse events**	**Control Fluid**	**Citrate Fluid**	**Wash out**
Total Adverse events	18	16	5
General disorders	*3*	*2*	*4*
Infections and infestations	*3*	*8*	*1*
Investigations	*2*	*0*	*0*
Metabolism and nutrition disorders	*3*	*0*	*0*
Others	*7*	*6*	*0*
Total serious adverse events (n)	5	3	2
Total product related adverse events (n)	0	3*	1**

* Classified as not assessable in relationship to study product by investigator but worst-case assessed by sponsor
** Patient accidentally received study product during wash-out

TABLE S2: Biocompatibility parameters (pre-dialysis)

	Control Fluid		Citrate Fluid	
**Parameter,** Mean ±SEM	**Week 0**	**Week 6**	**Week 0**	**Week 6**
Inflammation				
CRP (mg/l)	17.5 ±8.9	8.4 ±2.1	8.3 ±3.6	13.8 ±0.34
Pentraxin 3 (ng/ml)	1.76 ±0.29	1.90 ±0.30	1.82 ±0.39	2.01 ±0.34
IL-6 (ng/l)	17.3 ±8.2	11.4 ±1.7	11.9 ±2.3	13.6 ±3.0
TNF-α (ng/l)	26.3 ±1.3	24.9 ±1.5	26.3 ±1.4	25.5 ±1.4
Oxidative stress				
MDA (µM)	1.64 ±0.15	1.55 ±0.15	1.59 ±0.14	1.57 ±0.14
8-oxo-dG (ng/ml)	0.59 ±0.04	0.62 ±0.04	0.61 ±0.04	0.65 ±0.04
mAOPP (µM)	118.0 ±6.2	109.1 ±4.6	108.6 ±5.1	106.9 ±4.0
Glutathione GSH/GSSG ratio	0.28 ±0.04	0.28 ±0.04	0.32 ±0.04	0.25 ±0.03
γGluCys (µM)	3.10 ±0.16	3.08 ±0.16	3.21 ±0.18	3.01 ±0.16
CysGly (µM)	57.3 ±3.4	58.2 ±3.4	57.2 ±3.4	55.6 ±3.0
Cys (µM)	428 ±22	446 ±21	446 ±23	424 ±20
HomoCys (µM)	22.5 ±2.1	21.2 ±1.3	22.5 ±1.7	21.1 ±1.4
Advanced Glycation End-products				
Total AGE (AU)	6456 ±365	6280 ±326	6935 ±826	6466 ±293
CML (ng/ml)	122 ±9.8	116 ±10.2	119 ±9.6	118 ±10.1
Pentosidine (nM)	1489 ±154	1507 ±149	1468 ±144	1490 ±138
